# Supplementary figures and images for: Secuer: Ultrafast, scalable and accurate clustering of single-cell RNA-seq data
Source: PLoS Comput Biol. 2022 Dec 5;18(12):e1010753. doi: 10.1371/journal.pcbi.1010753 (PMC9754601; doi:10.1371/journal.pcbi.1010753)

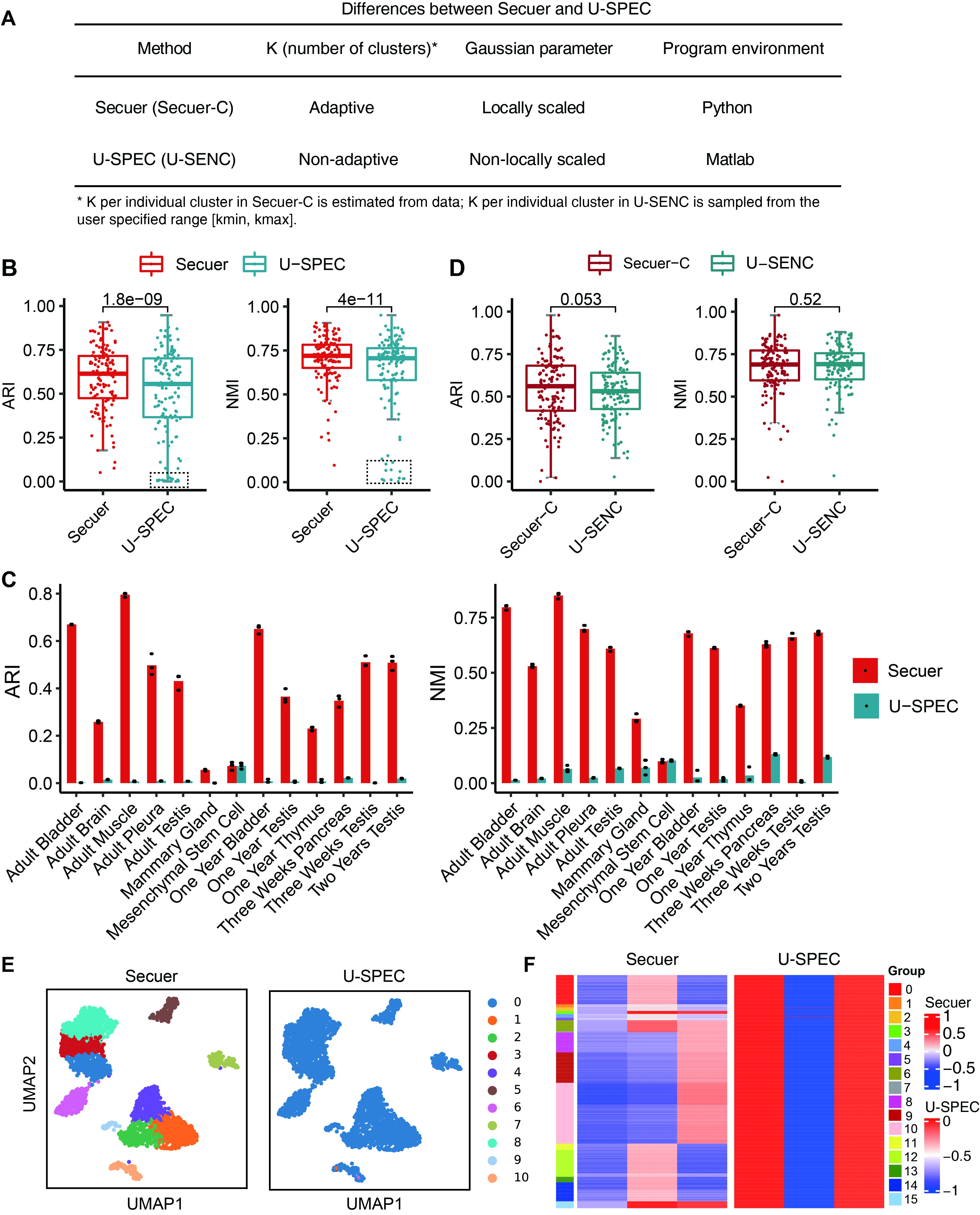

Supplement: S1 Fig — (A) The differences between Secuer and U-SPEC. Here the number of clusters K in Secuer is estimated from data (i.e., data-adaptive) and in U-SPEC is user-specified (i.e., not data-adaptive). (B) ARI (left) and NMI (right) of Secuer and U-SPEC on 128 datasets from Mouse Cell Atlas, where Secuer used a locally scaled Gaussian kernel and U-SPEC used a non-locally scaled Gaussian kernel. The detailed information on these datasets is provided in S2 Table. Each point is the average over 10 runs and the dashed rectangles refer to the datasets with poor results (defined as those with ARI < 0.1). P-values are computed from paired Wilcoxon test. (C) Barplots of ARI (left) and NMI (right) compare the performance of Secuer and U-SPEC in those datasets with poor results identified in (B). (D) ARI (left) and NMI (right) of Secuer-consensus (i.e., Secuer-C) and U-SENC. Here U-SENC is the consensus clustering method based on U-SPEC. (E) The UMAP of clustering results by Secuer (left) and U-SPEC (right) on the Adult bladder dataset. (F) Heatmap showing the eigenvectors of the bipartite graph Laplacian of Secuer and U-SPEC on the Adult bladder dataset, where rows represent cells and columns represent the eigenvectors corresponding to the top 3 largest eigenvalues. The ground-truth labels are plotted as Group. (TIF) [file pcbi.1010753.s001.tif]

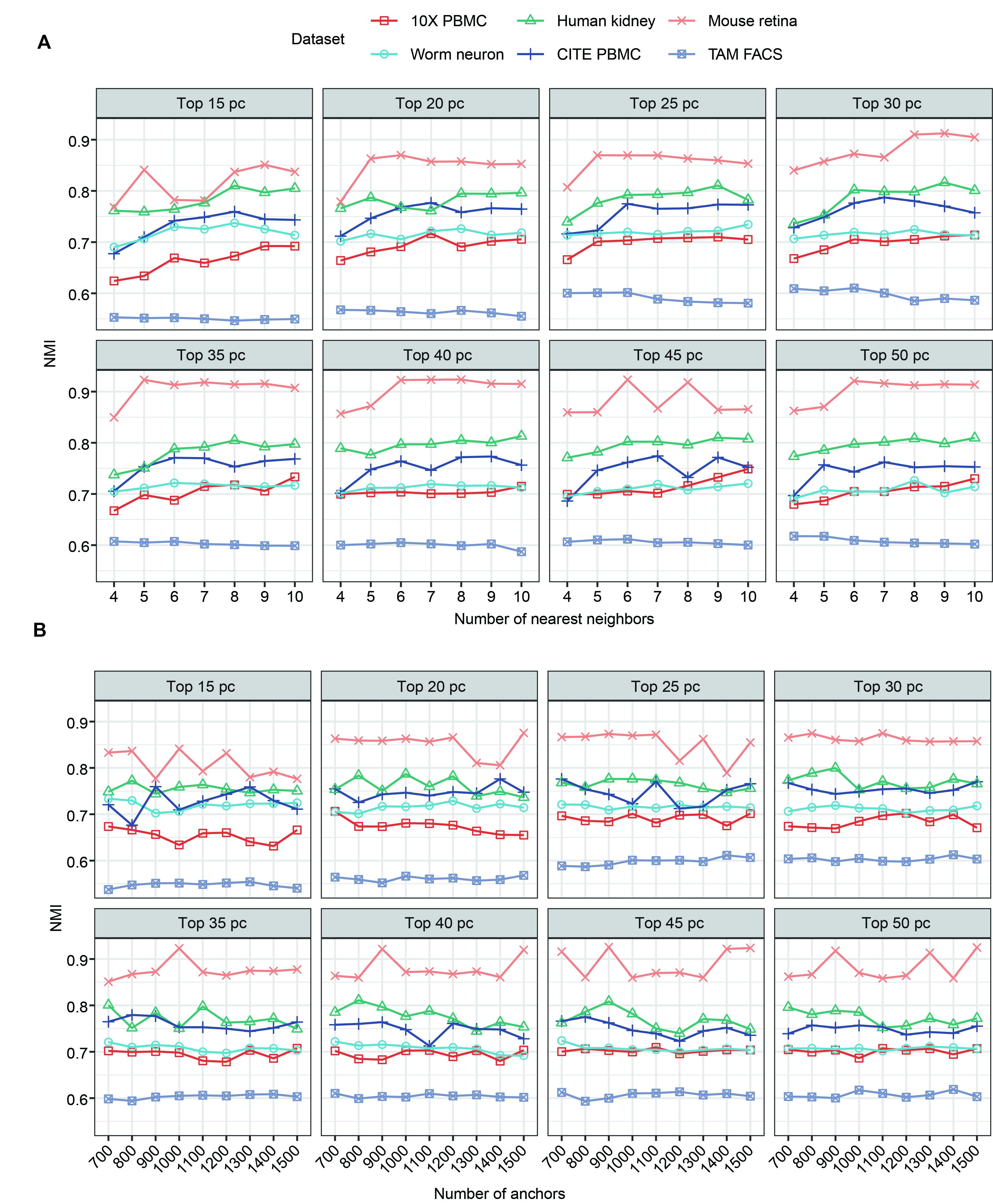

Supplement: S2 Fig — (A) The NMIs for six datasets are computed over different top numbers of principal components (pc) and different numbers of nearest neighbors in MAKNN. (B) The NMIs for six datasets are computed over different numbers of principal components and different numbers of anchors. Different panels represent different numbers of principal components. (TIF) [file pcbi.1010753.s002.tif]

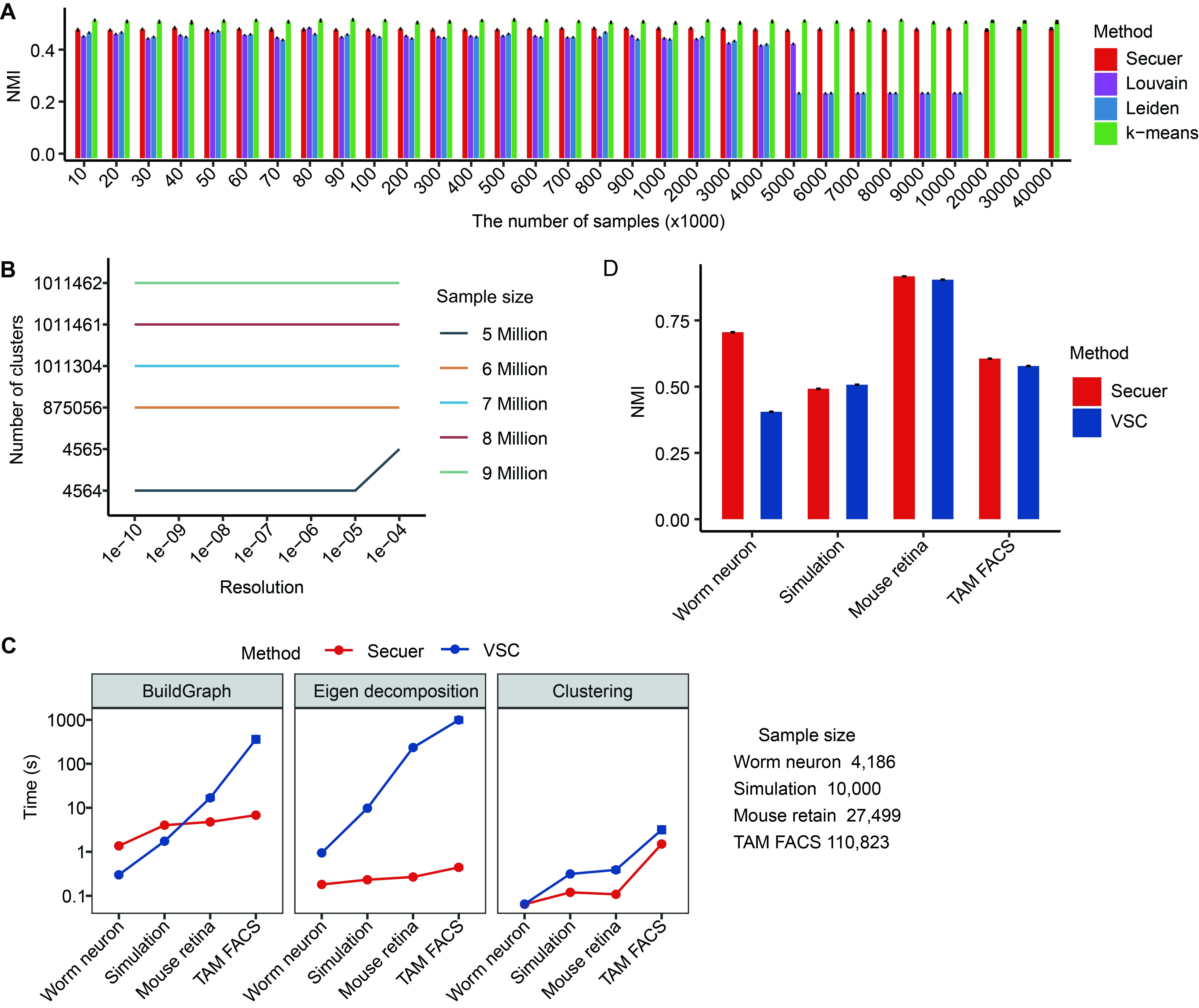

Supplement: S3 Fig — (A) The NMI of different methods on simulated datasets with different sample sizes. The simulated datasets with an increasing number of cells ranging from 10,000 to 40 million are generated from Mouse brain datasets (see Materials and Methods for more details). (B) The number of clusters estimated by Louvain in five simulated datasets with sample sizes ranging from 5 million to 9 million under different resolutions (x-axis). (C) We divided the entire clustering procedure into three steps and showed the runtime of each step taken by Secuer and vanilla spectral clustering (VSC) on four datasets, including Worm neuron, Simulation data with 10,000 samples, Mouse retina and TAM FACS with the number of cells ranging from 4,217 to 110,823. (D) The NMI of two methods on the four datasets. (TIF) [file pcbi.1010753.s003.tif]

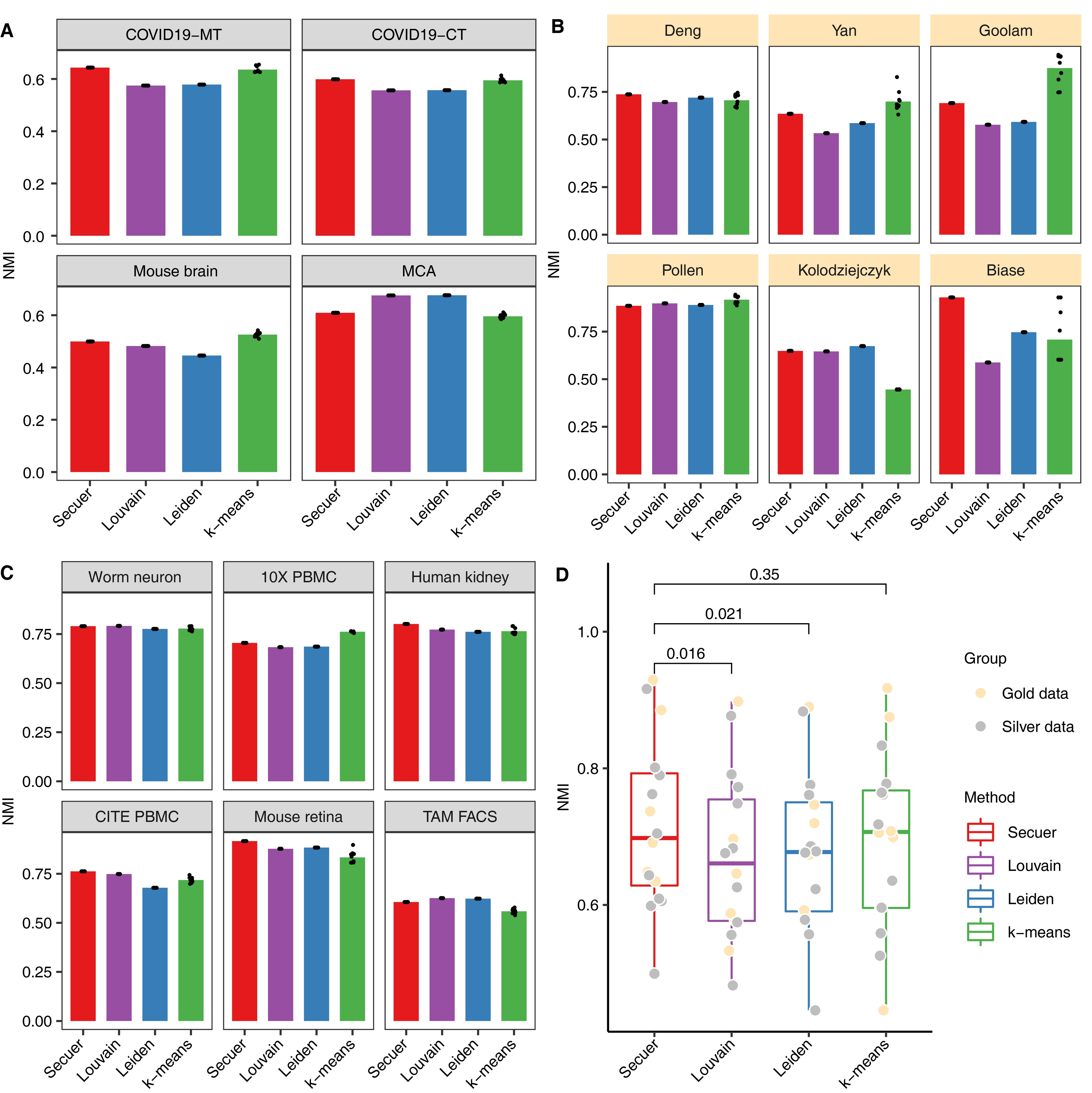

Supplement: S4 Fig — (A-C) The NMI of the different methods on the three large datasets (A), six gold standard datasets (B) and six silver standard datasets (C), where the COVID19-MT refers to the major cell types label, and COVID19-CT refers to the cell types label provided by author in COVID19 dataset. (D) The summary of NMI of the different methods on 15 datasets. (TIF) [file pcbi.1010753.s004.tif]

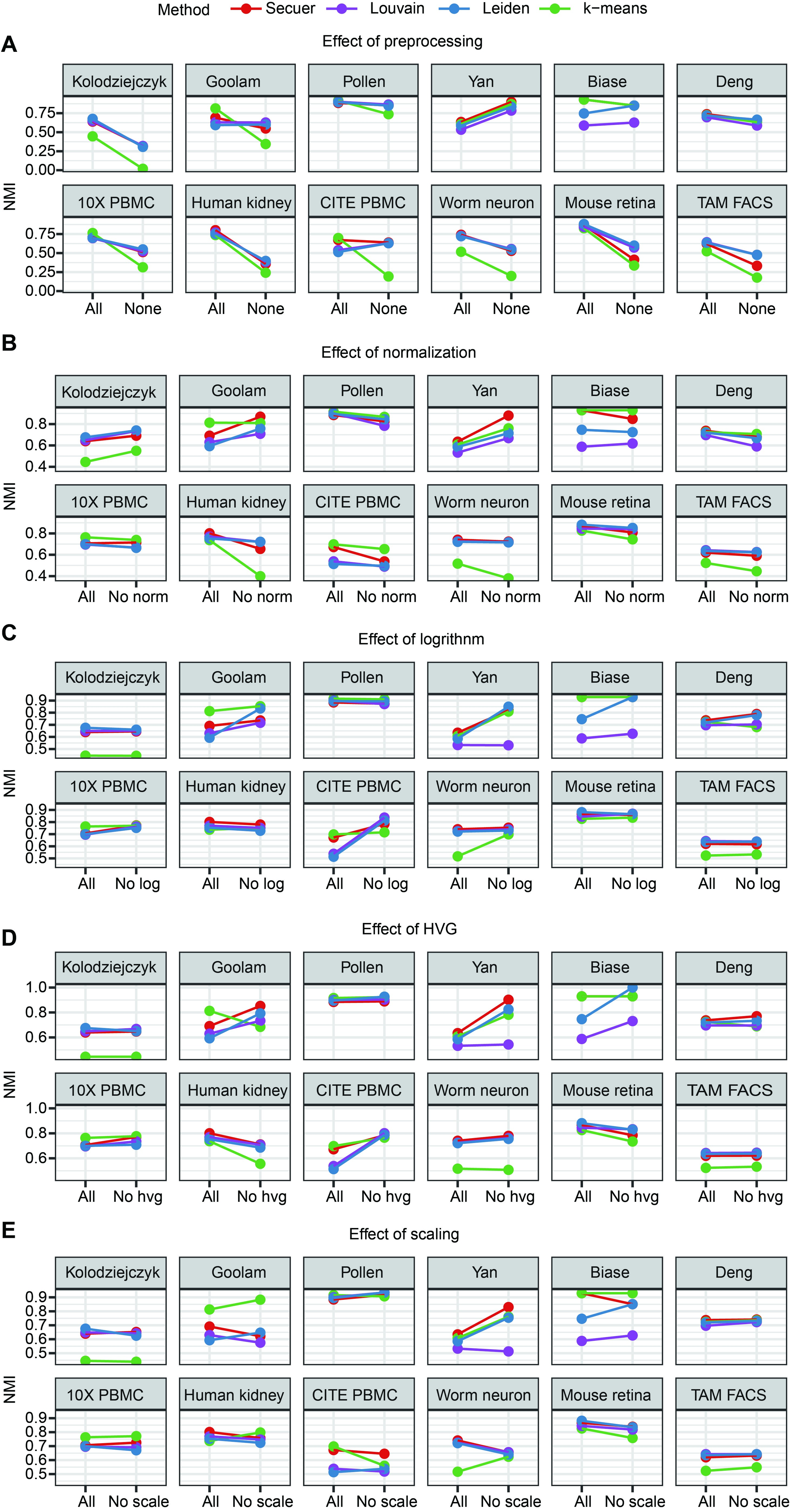

Supplement: S5 Fig — (A) Clustering performance with and without preprocessing, in which "ALL" refers to using all four preprocessing steps including normalization, logarithmic transformation, selection of high variable genes (HVG) and scaling (zero mean and equal variance), and ‘None’ refers to omitting all four steps. (B-E) Comparison of clustering accuracy between removing one of the steps and ‘ALL’, including removing normalization (B) logarithmic transformation (C), selection of high variable genes (D) and scaling (E). (TIF) [file pcbi.1010753.s005.tif]

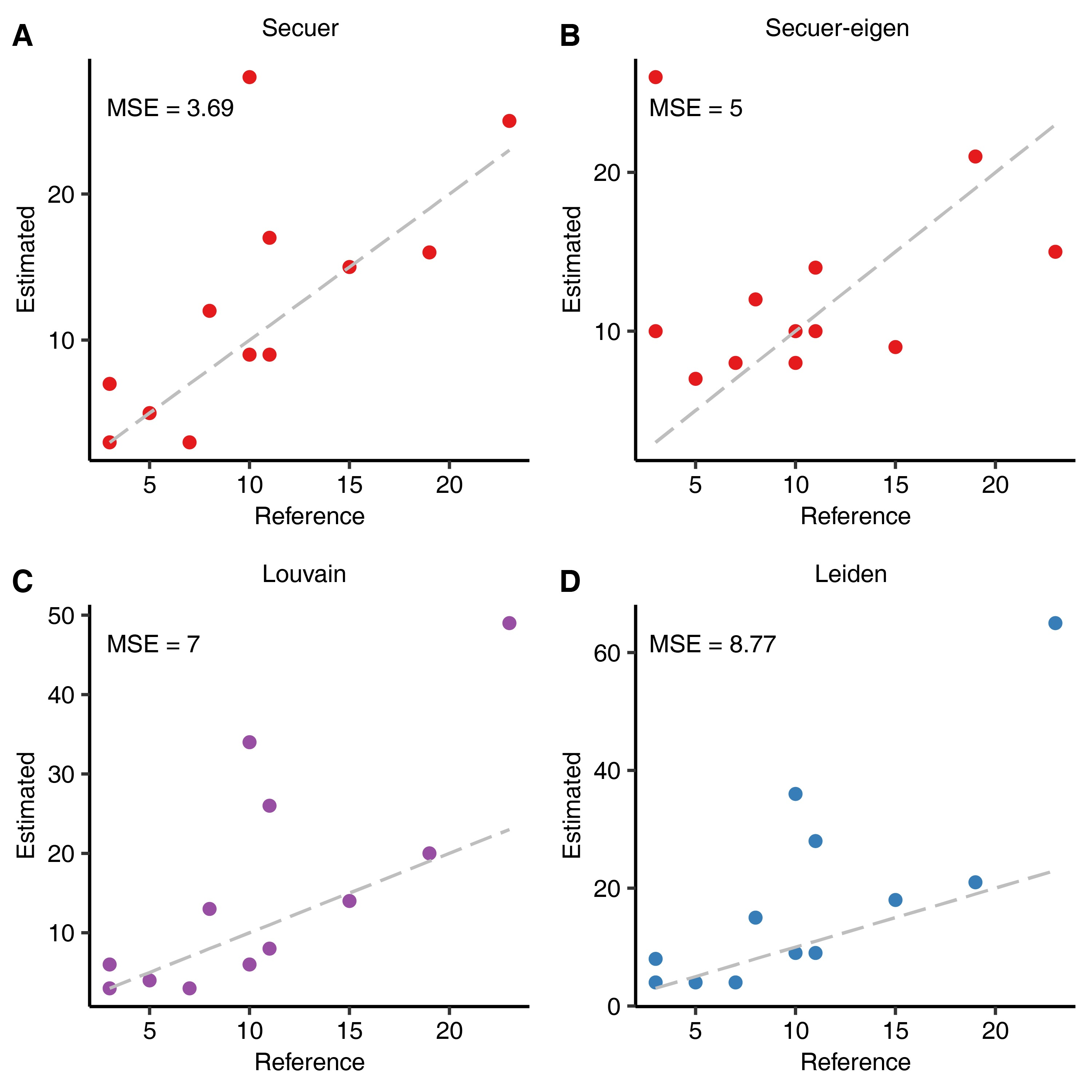

Supplement: S6 Fig — (A-D) The Pearson correlation between the estimated number of clusters and the ground-truth (Reference) across different methods: Secuer: based on the community detection on the anchor graph (A), Secuer-eigen: based on the eigenvalues of bipartite graph Laplacian between cells and anchors (B) (see Materials and Methods), Louvain (C), and Leiden (D). The mean absolute error (MSE) for each method is shown in the top left corner of the plot. (TIF) [file pcbi.1010753.s006.tif]

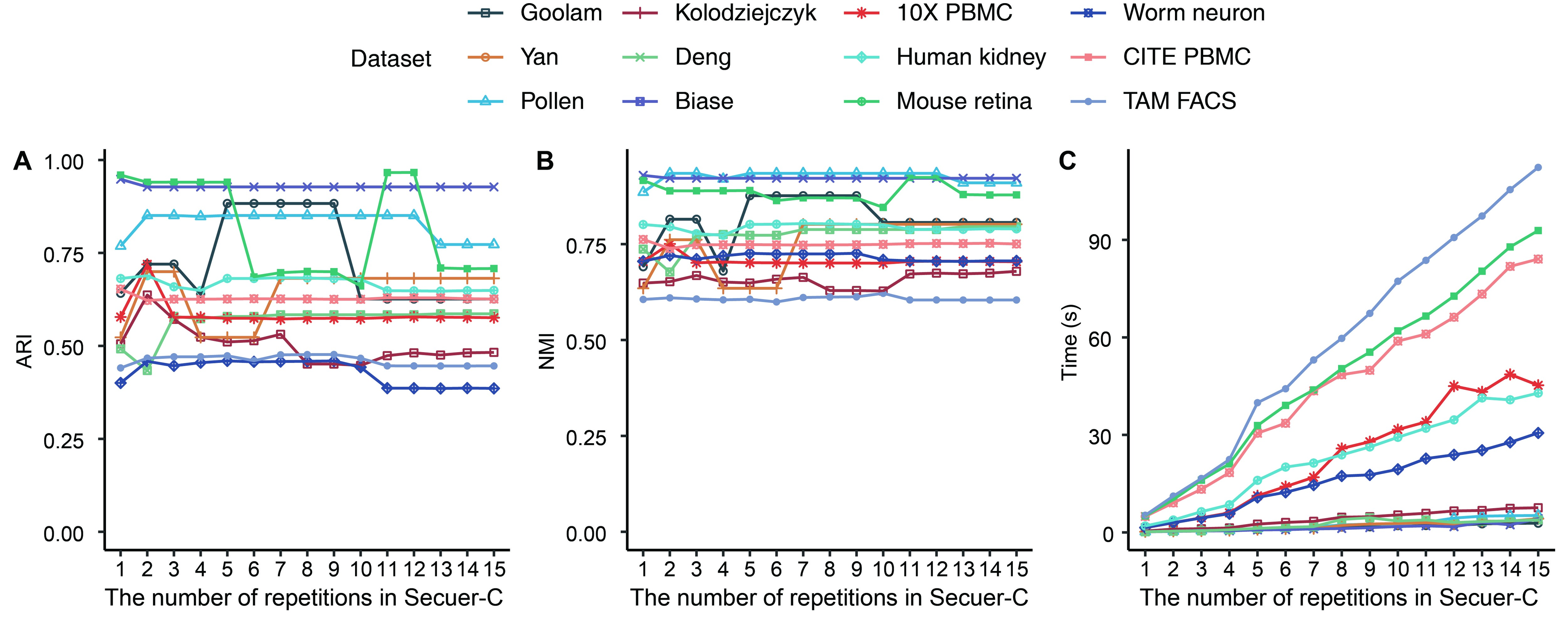

Supplement: S7 Fig — (A-C) Clustering accuracy quantified by ARI (A) and NMI (B) vs. the number of repetitions in consensus clustering (M) outputs of Secuer fed into Secuer-consensus (i.e., Secuer-C) over different datasets. (C) Runtime vs. M over different datasets. (TIF) [file pcbi.1010753.s007.tif]

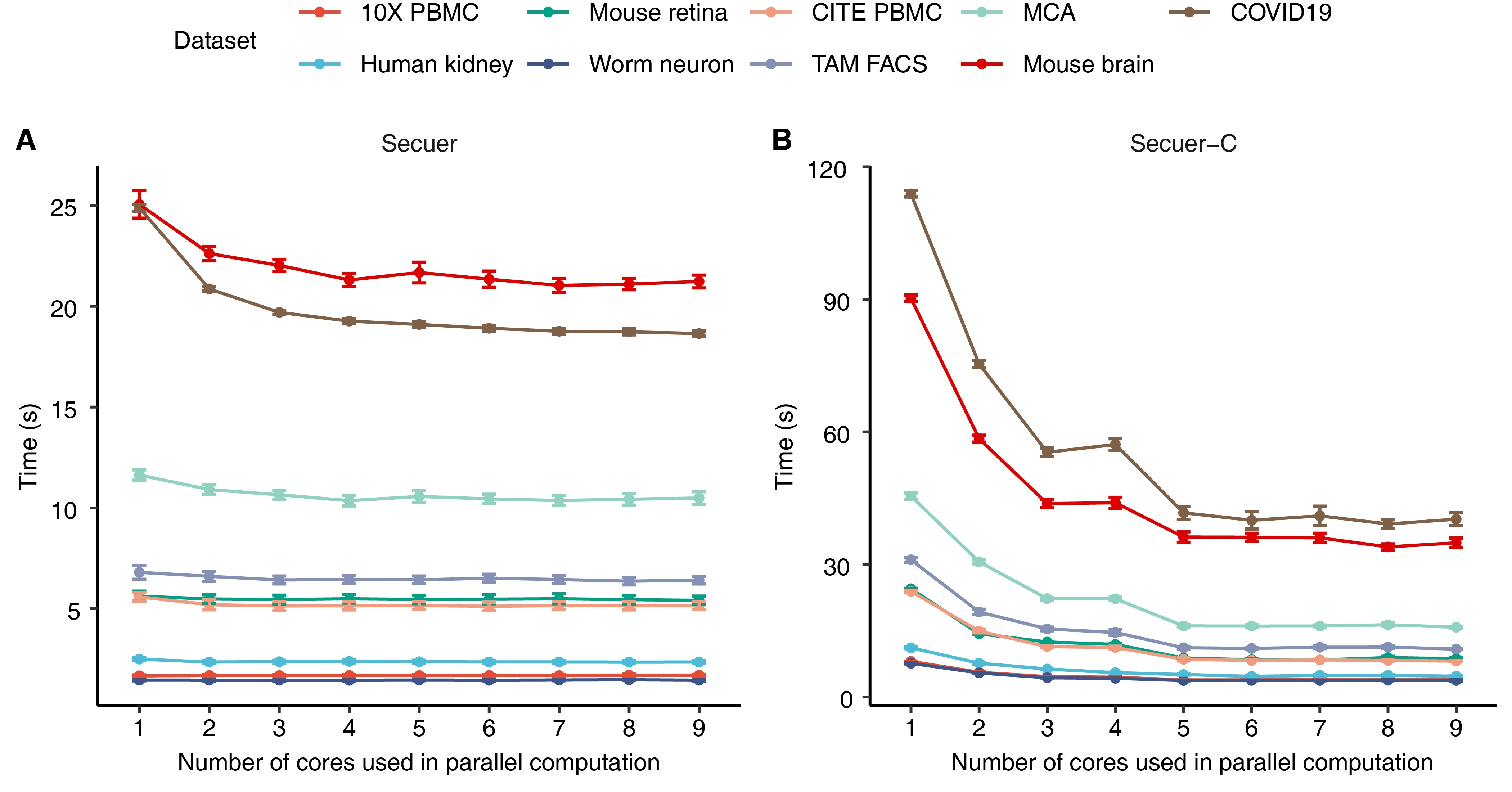

Supplement: S8 Fig — (A) Clustering time of Secuer (A) and Secuer-consensus (i.e., Secuer-C) (B) vs. the number of cores used in parallel computation on different datasets. (TIF) [file pcbi.1010753.s008.tif]
