# Supplementary material for: Secuer: Ultrafast, scalable and accurate clustering of single-cell RNA-seq data
Source: PLoS Comput Biol. 2022 Dec 5;18(12):e1010753. doi: 10.1371/journal.pcbi.1010753 (PMC9754601; doi:10.1371/journal.pcbi.1010753)
Supplement: Attachment — Submitted filename: Response-Letter.docx [file pcbi.1010753.s011.docx]

We would like to thank the editor and two anonymous reviewers for their insightful review and constructive suggestions, also for the positive comments such as “utmost clarity”, “simplicity”, and “rigor” on our work. Based on the editor and reviewers’ suggestions, we have carefully revised our manuscript (modifications in the manuscript are highlighted in yellow).

In summary, we have mainly made the following changes.

1.	We have compared the performance of U-SPEC and Secuer on 128 single cell datasets from Mouse Cell Atlas. The results are displayed in **S1 Fig**. Generally, Secuer has better performance than U-SPEC in these scRNA-seq datasets because unlike U-SPEC, Secuer implemented a locally weighted Gaussian kernel, which can adapt to the dataset-specific local geometry.

2.	We have accelerated the runtime of our method using parallel computation. The results are shown in **S8 Fig**.

We feel that these additional analyses have notably enhanced the quality and clarity of the manuscript. We hope, together with other minor changes, the current version of our paper has addressed all points raised by the reviewers. Please find enclosed our detailed responses to each of the reviewer’s specific points as well as the revised manuscript, figures and supplement. (The reviewers’ comments are in blue, and our replies are in black.)

**Reviewer #1:**

This paper presents a bipartite graph-based clustering algorithm for single-cell RNA-seq clustering. This work is similar to the U-SPEC algorithm in [23]. Please provide more detailed and specific discussions about the similarity and dissimilarity between the proposed algorithm and the U-SPEC algorithm. Experimental comparison between the proposed algorithm and the U-SPEC algorithm is also suggested.

In the experiments, the benchmark datasets and the baseline algorithms should be described with more details. An additional table of their statistics can help improve the clarity.

In the reference [23], multiple U-SPEC clusters are jointly modeled via an ensemble clustering strategy. Similarly, some ensemble clustering techniques, such as multidiversified ensemble clustering (MDEC) and enhanced ensemble clustering via fast propagation of cluster-wise similarities, can also be considered in the future extensions.

**Response:**

Thank you for raising these points. We have now explained the differences between Secuer and U-SPEC in the Introduction (lines 60-61) and **S1A Fig** and assessed the performance of both methods using 128 scRNA-seq datasets covering diverse tissues from Mouse Cell Atlas (**S1B-1F** **Fig**). In short, these results demonstrate that Secuer performs slightly better than U-SPEC.

Specifically, there are three major differences between Secuer and U-SPEC (**S1A** **Fig**). First, our method can adaptively determine the number of clusters ($K$), while U-SPEC requires users to specify $K$ (non-adaptive). For the consensus clustering methods, the number of clusters ($K$) per individual clustering in Secuer-C is also estimated from data (adaptive), while in U-SENC it is sampled from some user-specified range [kmin, kmax] (non-adaptive). Second, we employ a locally scaled Gaussian kernel to measure the similarity between cells and anchors to increase the clustering accuracy and robustness (see below), while U-SPEC uses a non-locally weighted Gaussian kernel. Finally, Secuer is implemented in Python, and U-SPEC is in Matlab.

As shown in **S1B Fig**, our method generally outperforms U-SPEC. In addition, in some datasets, U-SPEC has very low clustering accuracy (demonstrated in dashed rectangle in **S1B Fig**). The ARI/NMI of these individual datasets are shown in **S1C Fig**.

Taking the Adult bladder dataset as an example, U-SPEC classifies nearly all cells to a single cluster, while clustering results of Secuer agree with the UMAP graph (**S1E Fig**). In **S1F Fig**, we provided the eigenvectors of the bipartite graphs from the two methods on this dataset, with rows representing cells and columns representing the eigenvectors. The ground-truth labels are plotted as Group. The eigenvectors from U-SPEC are the same for different cells leading to the poor clustering performance, which provided evidence that the locally scaled Gaussian kernel used in Secuer is more suitable to capture the local geometry of single cell gene expression profiles, at least for some of the datasets evaluated in this paper.

For the consensus method, Secuer-C performs slightly better than U-SENC in terms of ARI, and has similar performance to U-SENC in terms of NMI (**S1D Fig**). However, it is worth emphasizing that in these experiments, we provided the TRUE number ($K$) of clusters while running U-SENC in the consensus step, while Secuer-C automatically estimates $K$ from the data. Hence we believe the slightly better performance of Secuer-C is even more convincing.

Finally, we are very grateful for the suggestion of implementing MDEC in the future extensions of Secuer.

**Reviewer #2:**

The authors propose a sound and elegant clustering approach that shows notable (time and memory) efficiency for massive data while remaining accuracy-wise competitive with state-of-the-art alternatives. To this end, Secuer relies on simplistic yet highly effective principles: pivoting of anchors for scaling up efficiency, anchor neighborhood per observation, locally-scaled Gaussian kernel on the neighborhoods to better capture observation-to-anchor similarity, and automated estimation of the number of clusters.

The manuscript is written with outmost clarity, simplicity, and rigor.
The experiments are comprehensive and appropriate to assess the bold claims. Comparison against state-of-the-art approaches, as well sensitivity analysis on the parameters, is conducted across a large base of dataset, including diverse scRNA-seq datasets, complementary large real-world datasets, well-annotated benchmarks, and synthetic data.

The software is provided as an open-source module in GitHub, ensuring the reproducibility of the acquired results.

**Response:**

We appreciate the reviewer’s positive comments on our manuscript.

**Some minor-moderate concerns:**

1) consider moderating a few claims:
- "Secuer enjoys reduced runtime and memory usage by orders of magnitude" -> over one order of magnitude for datasets with more than 1 million cells
- "again greatly improves"

-We have rewritten “Secuer enjoys reduced runtime and memory usage by orders of magnitude, especially for ultra-large datasets profiling over 1 or even 10 million cells” as “Secuer enjoys reduced runtime and memory usage over one order of magnitude for datasets with more than 1 million cells.” in Abstract.
-We removed the “greatly” of “again greatly improves” in Abstract.

2) it is not always clear what is the default estimator to approximate the number of clusters in Secuer, please clarify whether you opted to use the Louvain estimator on the anchors or the near-zero eigenvalue method. It is also not completely clear whether the later method was assessed.
In addition, consider disclosing in the overview the selected community detection method, as well as adding reference [11] after "near-zero eigenvalues of the graph Laplacian (see Materials and Methods)".

We have described the default method used in Secuer in Overview of Results and Materials and Methods sections (lines 363-364), and have added reference [11] in Overview of the Results section.

3) to fully attest the scalable nature of the proposed approach, consider further assessing whether Secuer can be parallelized/distributed and, if so, introducing high-level principles to this end

We have demonstrated that our method can be accelerated by using parallel computation and have reported the results in **S8 Fig**. The average runtime is decreased by 50% on datasets with more than 1 million cells using 3 cores.

4) the intuition for the locally-scaled Gaussian kernel is a simple one, please briefly provide it in the Overview instead of referencing “Materials and Methods”.

We have added a brief introduction of the locally-scaled Gaussian kernel in the Results section under Overview of Secuer as “…, that the bandwidth parameter of each cell is defined as the average distance between the cell and its $k$ nearest neighbor anchors, …”.

5) "The preprocessing involves four steps: 1) gene/cell filtering; 2) normalization; 3) selection of highly variable genes; 4) dimension reduction by PCA" -> please ensure that the applied preprocessing parameters are available for all the tested datasets as they critically impact experiments.

We have revised the manuscript to clarify this point and provided the preprocessing code to improve the reproducibility of our work. Specifically, we have added the sentence “The parameters of the preprocessing pipeline are the same for all datasets except for the gene/cell filtering step, which instead follows the criteria in the original studies… Scripts for all the above preprocessing steps are available at https://github.com/nanawei11/Secuer.” to the Materials and Methods section (lines 483-484, 498-499).

6) minor notes on complexity majorants:
- (2) -> please introduce d and N right after or at the beginning ('Let d and N be...')
- (1/2) -> provide the intuition for the squared root of 'p' in the Materials and further disclose the meaning of 'o' parameter

-In the first paragraph of the Materials and Methods section, we have now defined $N$ and $d$ right after the first time that they were mentioned. We have also tried to remind readers the meanings of the notation whenever we feel appropriate.
-We have (1) explained the reason for choosing $o$ to be $p^{\frac{1}{2}}$ and (2) illustrated the meanings of $p$and $o$ in the Materials and Methods section under “The MAKNN algorithm” (lines 394-396).

7) proof-check for minor language aspects:
- method Secuer for -> method, Secuer, for
- Fig1F -> Fig 1F
- "It appears that" -> consider instead referencing memory 'estimates' to avoid 'appears'
- "divide the graph into disjoint subgraphs" -> please validate the use of 'disjoint'.

Thank you for pointing out these issues/typos. We have revised “Fig1F” to “Fig 1F”, “It appears that” to “We observed that” and “disjoint” to “connected”.

8) few notes on the supplementary material
- reformat S1
- no information is provided on the simulated datasets in S3, please clarify the generation procedure
- consider starting from a lower number of solutions in S7 to better assess the impact of consensus

-We have renamed the S1 as **S1 Table** (see the Supplementary files).
-We have illustrated the source of the simulated datasets in **S3 Fig**.
-We have added the consensus runtimes from 1 (no consensus) to 15 in **S7 Fig**.
